# Stevens' Hospital, Dublin

**Published:** 1832-11

**Authors:** 


### STEVENS' HOSPITAL, DUBLIN.

*Case of Compound Fracture of the Skull, treated by* Mr. Cusack.
[*From a Paper by* Mr. Crampton, *on Injuries of the Head. Dublin Journal of Med. and Chem. Science.*]

James Fagan, aged twenty-three, a tall and powerful man, rather full than otherwise, admitted at twelve o'clock of the night of the 17th of March, 1832. received a severe wound, five inches long, from a dragoon's sword (convex on its cutting edge) over the right parietal bone. The sword had cut through the bone and membranes of the brain, as some cerebral substance appeared on the lips of the wound. When admitted, he was violent and incoherent, roared very much while being dressed, answered questions with reluctance, and then incoherently; vomited twice, skin warm, pulse ninety-two, and weak. The wound was inflicted about half an hour before his admission. When struck, he fell forwards, and lay insensible for five minutes, then leaped up, made a rush at the door, as if to escape, but fell again. He had been drinking for some hours when he received the wound.

March 18. Present state. He lies on his side as if asleep; his eyes closed; when roused and asked a question, he moans and answers peevishly, but often intelligibly. His whole aspect and manner is that of a person oppressed with drink and sleep. When much roused, he can tell where he received the injury, but not how. Says he has a pain in his head, points to the wound with his hand, and immediately relapses into a state resembling tranquil sleep. Surface of the body warmer than natural; pulse eighty-eight, small and variable, becoming rapid when he moves, or is raised in the bed; tongue moist, but brown in the centre. Pupils of equal size, contract on exposure to light; has some cough, which seems to distress him very much, for when it attacks him, he grasps the bed with both hands and groans with agony; has vomited four or five times during the night. On removing the dressing this morning, a good deal of cerebral substance mixed with blood escaped from the wound.—℥xvi. of blood were taken from the arm. Ordered a bolus of Calomel and Jalap. Fever diet.

March 19. The bolus has not operated, was probably rejected by vomiting. A pint and a half of urine drawn off by the catheter last evening. Got some sleep; did not rave; is more sensible: answers when spoken to, but still with reluctance; would not allow the catheter to be passed this morning, but about a pint of high-coloured urine voluntarily passed afterwards. Pulse seventy-five, soft and regular. Tongue brown at the centre. Ordered a purging enema.

March 20. More torpid to-day; moans frequently; complains of pain in the head; pulse fifty-five when he lies quietly, but when he is disturbed it rises to eighty. Pupils natural; no stool; he will not shew his tongue.—℥xx. of blood taken from the arm. Pills with calomel and colocynth, and a cathartic enema. Wound glued together by lymph and pus.

March 21. Bowels well freed; stupor not so great; pulse sixty-eight; puts out his tongue when desired; passes his urine voluntarily; wound looks clean.—xxiv. leeches to the head; a cathartic enema. Two grains of calomel every three hours.

He continued much in the same state, but gradually becoming more sensible, until the 24th, when he appeared more torpid, and at four o'clock, P.M. he had a violent convulsive attack, four men could scarcely keep him in the bed; three hours afterwards he had another fit equally severe, which was followed by great stupor. As there were now evident symptoms of irritated, and probably compressd brain, it was determined, in consultation, by Mr. Crampton, Mr. Peile, Mr. Collis, Mr. Wilmot, and Mr. Cusack, to lay bare the wound in the skull freely, and then to act according to circumstances respecting the removal of a portion of the bone. As a considerable quantity of brain had escaped from the wound in the first instance, it was obvious that both tables of the skull, the membranes of the brain, and the brain itself, had been divided by the sword, and it was probable that the inner table of the skull had been separated from the outer, and driven in upon the brain: and such, upon a careful examination with the probe, seemed to be the case. A slip of bone about a quarter of an inch broad, and three inches long, was removed upon the upper edge of the divided bone, by means of a straight saw, about four inches in length: the operation was performed by Mr. Cusack, and was effected in a very few minutes, and with great ease. On removing the slip of bone, it was ascertained that the inner table was detached, so as to form an acute angle with the outer table. Some softened cerebral substance escaped from the wound; he had one slight convulsive attack after the operation, but he soon became much more sensible, and now (four hours after the operation) he lies tranquilly with his eyes open.

March 25. Had a convulsive attack at ten o'clock last night, but is quite free from stupor this morning; answers questions freely; pulse seventy-six, small and weak; complains of his head. Every thing went on favorably until the 27th of March, when a small red pulsating tumor, about the size of a pea, appeared in the centre of the wound.—Ordered ℥i. of infusion of Cinchona three times a day.

March 28. The fungus has doubled its size; is of a light purple colour, and pulsates slightly; wound granulating; general symptoms favorable; no material change occurred in the local or general symptoms, until the 13th of April, when the fungus began to enlarge considerably, and he became more torpid, and irritable; complained of pain in the head; the fungus pulsates strongly, and is as large as a small walnut. About the 16th of April the fungus began to decline rapidly in size; the general symptoms at the same time became more favorable. On the 20th the fungus had disappeared, and about the 1st of May the wound was completely healed.

Fagan was discharged from the hospital on the 15th, in good health, but his memory was much impaired; he was able, however, to resume his work as a pipe-maker. I saw him this 20th of July, 1832; his health is excellent, but his memory of words, but not of things, is greatly impaired; he told me "he knew every thing as well as ever he did, but he could not put a name on any thing." I shewed him a button, he laughed, and said "I know what it is very well, it is a ba, ba, ba,—Och! I can't say it, but there it is," pointing to a button on his own coat.

*Observations.* I think it probable that, had the portion of depressed bone been removed in this case in the first instance, namely, the 17th of March, or had the operation been delayed until the 25th or 26th of March, the result would not have been so favorable. The operation, in the first instance, would [not] have been an additional violence to parts already severely irritated, and consequently an additional source of inflammation; it would besides have removed all support from the wounded brain, a great part of which would (it is probable) have escaped through the opened dura mater. If the patient escaped these first dangers, then came the danger of hernia, or rather fungus cerebri, one of the most frequent and dangerous consequences of wounds of the dura mater. Had the operation been postponed even for a few hours after decided symptoms of cerebral irritation (as evinced by the convulsions) had set in, it would have but aggravated the mischief, by irritating parts already in a state of incipient inflammation. That the operation may be resorted to with advantage, even after the appearance of symptoms, which, if not relieved, almost invariably proceed to a fatal termination, is an important fact which has not perhaps been sufficiently insisted upon.

